# Structures of Coxsackievirus A16 Capsids with Native Antigenicity: Implications for Particle Expansion, Receptor Binding, and Immunogenicity

**DOI:** 10.1128/JVI.01102-15

**Published:** 2015-08-12

**Authors:** Jingshan Ren, Xiangxi Wang, Ling Zhu, Zhongyu Hu, Qiang Gao, Pan Yang, Xuemei Li, Junzhi Wang, Xinliang Shen, Elizabeth E. Fry, Zihe Rao, David I. Stuart

**Affiliations:** a Division of Structural Biology, University of Oxford, The Henry Wellcome Building for Genomic Medicine, Headington, Oxford, United Kingdom; b National Laboratory of Macromolecules, Institute of Biophysics, Chinese Academy of Science, Beijing, China; c National Institutes for Food and Drug Control, No. 2, Tiantan Xili, Beijing, China; d Sinovac Biotech Co., Ltd., Beijing, China; e National Vaccine and Serum Institute, Beijing, China; f Laboratory of Structural Biology, School of Medicine, Tsinghua University, Beijing, China; g Diamond Light Sources, Harwell Science and Innovation Campus, Didcot, United Kingdom

## Abstract

Enterovirus 71 (EV71) and coxsackievirus A16 (CVA16) are the primary causes of the epidemics of hand-foot-and-mouth disease (HFMD) that affect more than a million children in China each year and lead to hundreds of deaths. Although there has been progress with vaccines for EV71, the development of a CVA16 vaccine has proved more challenging, and the EV71 vaccine does not give useful cross-protection, despite the capsid proteins of the two viruses sharing about 80% sequence identity. The structural details of the expanded forms of the capsids, which possess nonnative antigenicity, are now well understood, but high resolution information for the native antigenic form of CVA16 has been missing. Here, we remedy this with high resolution X-ray structures of both mature and natural empty CVA16 particles and also of empty recombinant viruslike particles of CVA16 produced in insect cells, a potential vaccine antigen. All three structures are unexpanded native particles and antigenically identical. The recombinant particles have recruited a lipid moiety to stabilize the native antigenic state that is different from the one used in a natural virus infection. As expected, the mature CVA16 virus is similar to EV71; however, structural and immunogenic comparisons highlight differences that may have implications for vaccine production.

**IMPORTANCE** Hand-foot-and-mouth disease is a serious public health threat to children in Asian-Pacific countries, resulting in millions of cases. EV71 and CVA16 are the two dominant causative agents of the disease that, while usually mild, can cause severe neurological complications, leading to hundreds of deaths. EV71 vaccines do not provide protection against CVA16. A CVA16 vaccine or bivalent EV71/CVA16 vaccine is therefore urgently needed. We report atomic structures for the mature CVA16 virus, a natural empty particle, and a recombinant CVA16 virus-like particle that does not contain the viral genome. All three particles have similar structures and identical antigenicity. The recombinant particles, produced in insect cells (a system suitable for making vaccine antigen), are stabilized by recruiting from the insect cells a small molecule that is different from that used by the virus in a normal infection. We present structural and immunogenic comparisons with EV71 to facilitate structure-based drug design and vaccine development.

## INTRODUCTION

Enterovirus 71 (EV71) and coxsackievirus A16 (CVA16) are subgroup A human enteroviruses (HEVA) (1), small, nonenveloped single-stranded RNA (ssRNA) viruses whose protective capsid mediates cell entry and humoral immune responses. The external portion of the icosahedral capsid comprises 60 copies of viral proteins VP1, -2, and -3 arranged with pseudo T=3 symmetry, while their N-terminal extensions and 60 copies of VP4 line the interior, surrounding the RNA genome. Natural empty particles (without RNA) are often also formed, in which the final RNA-mediated coat protein cleavage (between VP4 and VP2) is not made.

There is a “canyon” (a depression encircling the 5-fold axes) on the surface of enteroviruses (2) that often harbors the receptor binding site, and there is a fatty acid binding site below the canyon base, within the hydrophobic β-barrel core of VP1. Receptor binding tends to dislodge the fatty acid molecule, a prerequisite to a cascade of structural changes (3, 4) that ultimately leads to the release of the N terminus of VP1 (5) and VP4 (6) to form the expanded 135S intermediate, or A particle (6, 7). This particle is endocytosed (8) and at some point engages fully with the vesicle membrane to deliver the RNA to the cell, leaving an empty 80S, or B particle. Both 135S and 80S particles are antigenically distinct from the mature virion. While there is no evidence that the 135S particle can be converted back to the mature virus, at least a subset of the changes which occur are reversible, and thus there is evidence that mature virus particles can “breathe,” leading transiently to the partial externalization of internal polypeptides (9, 10). Expanded capsids have been visualized by electron microscopy (EM) (11–18) and crystallography (3, 19, 20). Remarkably, the crystal structure of the expanded 135S intermediate of CVA16 captured the N terminus of VP1 in egress from the particle (20).

For many picornaviruses, two types of viral particles (with and without RNA) are produced during a natural infection, which may be separated using continuous sucrose density gradient ultracentrifugation. In some cases, the purified empty particles are expanded, as noted above (3, 21); however, sometimes the full and empty particles are structurally very similar and are antigenically indistinguishable, e.g., for hepatitis A virus (22). It seems likely that empty particles are initially assembled with virus-like antigenicity and that mishandling, for instance, converts them to the expanded form. Clearly, empty particles with virus-like antigenic structure are of considerable interest for vaccine development.

EV71 and CVA16 are the dominant causes of hand-foot-and-mouth disease (HFMD) in East Asia and, thus, are jointly responsible for millions of infections and hundreds of deaths. They are closely related (∼80% sequence identity in the capsid proteins), and both of them utilize human P selectin glycoprotein ligand 1 (PSGL-1) or human scavenger receptor class B, member 2 (SCARB2) receptors (23, 24); however, EV71 vaccine at best elicits very weak cross-protection against CVA16 (25–27). Based on their structures, it might be possible to identify cross-protective epitopes, which would be useful for a more widely effective HFMD vaccine. We report here high resolution crystal structures for both mature and natural empty CVA16 formaldehyde-treated particles and baculovirus-expressed recombinant capsids (viruslike particles [VLPs]) and detail the differences between CVA16 and EV71 that determine their respective antigenicities.

## MATERIALS AND METHODS

### CVA16 production and purification.

CVA16 (genotype B), isolated in Zhejiang Provence, China, was produced in Vero cells (from the Shanghai Cell Bank of the Chinese Academy of Sciences) at a multiplicity of infection (MOI) of 0.25; the cells were cultured in Dulbecco's modified Eagle's medium (DMEM; Sigma) supplemented with 0.5% fetal bovine serum (FBS) (Gibco). Both cells and virus-containing supernatant were collected 5 days after infection, centrifuged to remove cell debris, ultrafiltered, and loaded for sucrose density gradient ultracentrifugation. CVA16 was inactivated by formaldehyde and purified as described previously (20). We have already shown that formaldehyde treatment does not significantly affect the structure of the closely related EV71 (3).

### Expression and purification of CVA16 VLPs.

A modified pFastBac Dual vector (Invitrogen) containing P1 (precursor of structural proteins) and 3CD (precursor of the 3C protease and 3D polymerase) genes under the control of the polyhedrin and cytomegalovirus (CMV) promoter, respectively, was kindly provided by P. Zhu (Institute of Biophysics, Chinese Academy of Sciences, Beijing, China) (28). Recombinant baculoviruses were generated using a Bac-to-Bac baculovirus expression system (Invitrogen) according to the instructions of the manufacturer. CVA16 VLPs were produced by infecting Sf9 insect cells (from the Shanghai Cell Bank of the Chinese Academy of Sciences) (2 × 10^6^ cells/ml) at an MOI of 10. The supernatants were collected 3 days after infection, centrifuged to remove cell debris, and filtered through a 0.22-μm filter (Millipore). Then, the supernatants were pelleted through a 30% sucrose cushion by ultracentrifugation in an SW28 rotor at 100,000 × *g* for 5 h at 4°C. Pellets were resuspended in phosphate-buffered saline (PBS) (pH 7.4), cleared by centrifugation at 10,000 × *g* for 5 min, loaded onto a 15%-to-45% (wt/vol) sucrose density gradient, and centrifuged in an SW41 rotor at 103,614 × *g* for 3.5 h at 4°C. Fractions containing VLPs were collected, dialyzed against PBS (pH 7.4), and concentrated for crystallization.

### Crystallization.

Crystallization primarily used nanoliter vapor diffusion in Greiner CrystalQuick X plates. Purified CVA16 mature and empty particles were concentrated to 2 and 3 mg/ml, respectively, in PBS (pH 7.4). Diamond-shaped crystals of both mature and empty CVA16 particles with a maximum size of 0.1 by 0.1 by 0.08 mm^3^ grew from the same condition (3.2 M sodium chloride, 0.1 M sodium acetate trihydrate, pH 7.0) within 2 weeks. Crystals of CVA16 VLPs were grown at a concentration of 3.5 mg/ml at 16°C using the hanging-drop vapor diffusion method over a reservoir of 1.8 M ammonium citrate dibasic, 0.1 M sodium acetate trihydrate (pH 5.0).

### Data collection and structure determination.

Diffraction data of CVA16 mature and empty particles were collected at room temperature (21°C) from crystals in crystallization plates (*in situ* data collection) using a previously reported method (29) at beamlines I24 and I03, Diamond Light Source (Didcot, United Kingdom). Diffraction images of 0.1° oscillation were recorded on Pilatus6M detectors using beam sizes of between 0.02 by 0.02 mm^2^ and 0.02 by 0.08 mm^2^ at I03 or 0.02 by 0.02 mm^2^ and 0.05 by 0.05 mm^2^ at I24 depending on the size of the crystals. On I24, the X-ray beam was homogenized with a 0.25-mm carbon plate and focused downstream from the crystal. Using a 0.1-s exposure time and 100% beam transmission, typically 4 to 8 useful images could be collected from a crystal. Diffraction data of CVA16 VLPs were collected at 100 K at beamline BL41 of the Spring 8 synchrotron in Japan. Crystals were soaked in solution containing 80% (vol/vol) reservoir solution and 20% (vol/vol) glycerol prior to flash cooling with liquid nitrogen (Table 1).

**TABLE 1:** Data collection and refinement statistics

Parameter	Value(s) for:		
	Mature virus	Empty particle	VLP
Data collection statistics			
No. of crystals (no. of positions)	72 (83)	55 (88)	1
Space group	*P*4_1_2_1_2	*P*4_1_2_1_2	*P*4_2_32
Cell dimensions (Å)	*a* = *b* = 491.2, *c* = 708.7	*a* = *b* = 491.4, *c* = 708.9	*a* = *b* = *c* = 347.9
Resolution range (Å)	50.0–2.65 (2.70–2.65)^*a*^	50.0–2.70 (2.75–2.70)	50.0–2.50 (2.59–2.50)
No. of unique reflections	1,705,020 (38,785)	1,437,314 (18,633)	244,135 (24,125)
*R*_merge_	0.519	0.535	0.217
*I*/σ(*I*)	1.5 (0.4)	1.4 (0.4)	11.3 (4.1)
Completeness (%)	69.6 (31.8)	62.1 (16.2)	100 (100)
Redundancy	2.1 (1.2)	1.8 (1.1)	12.7 (10.2)
Refinement statistics			
Resolution range (Å)	50.0–2.65	50.0–2.70	50.0–2.50
No. of reflections	1,665,283/8,403	1,411,807/7,095	244,027/12,014
*R*_work_/*R*_free_^*b*^	0.278/0.283	0.291/0.292	0.176/0.184
No. of atoms	6,662	6,446	6,492
Average B factor (Å^2^)	29	35	37
RMS deviations			
Bond length (Å)	0.010	0.009	0.013
Bond angle (°)	1.6	1.5	1.7

a Values in parentheses are for the highest-resolution shell.

b Note that the *R*_free_ value is of limited significance because of the considerable noncrystallographic symmetry.

**FIG 1:**
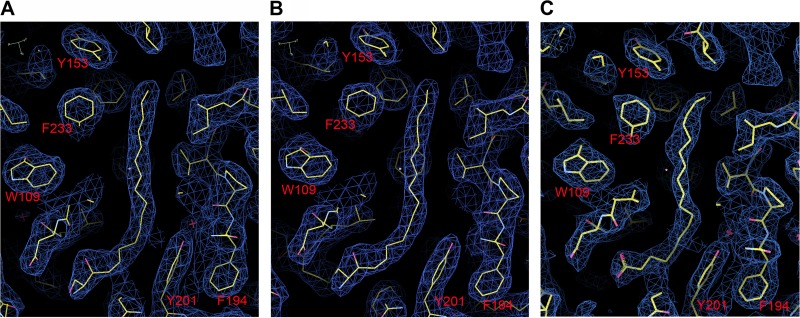
Electron density maps, showing electron density in the pocket factor binding region of VP1 in the full particles (A), natural empty particles (B), and recombinant VLPs (C) after NCS real space averaging.

Data were analyzed using HKL2000 (30), and structures determined by molecular replacement using MOLREP (31), with the coordinates of the mature EV71 particle (PDB accession number 3VBF) as the search model. Rigid-body refinement was followed by cyclic positional, simulated annealing, and B factor refinement used strict noncrystallographic symmetry (NCS) constraints with CNS (32). Averaging used GAP (DIS; J. Grimes and J. Diprose, unpublished data) and models were rebuilt with COOT (33) (Table 1; Fig. 1). Structural comparisons used SHP (34). Structural figures were prepared with PyMol (35). The coordinates and structure factors for the mature and natural empty CVA16 particles and CVA16 VLPs have been deposited with the RCSB under accession numbers 5C4W, 5C9A, and 5C8C.

### Production of monoclonal antibodies and neutralization assay.

Two groups of 6 adult (4 weeks old) female BALB/c mice (purchased from the Beijing Laboratory Animal Research Center) were immunized intraperitoneally with 5 μg of inactivated EV71 or CVA16 viruses, followed by two booster doses at 2-week intervals. Blood samples were obtained from the tails 2 weeks after the second booster and tested by enzyme-linked immunosorbent assay (ELISA), using purified EV71 and CVA16 viruses, respectively, as antigens. Monoclonal antibodies were produced using conventional protocols (continuous cultures of fused cells secreting an antibody of predefined specificity).

For the neutralization assay, purified monoclonal antibodies at a concentration of 0.2 mg/ml were initially diluted 8-fold as stocks and were then serially diluted 2-fold with DMEM containing 2% FBS. Amounts of 100 μl of 2-fold antibody dilutions were mixed with 100 μl of EV71 or CVA16 virus containing 100 50% tissue culture infective doses (TCID_50_) for 1 h at 37°C, and then the mixture was added to monolayers of Vero cells in 6-well plates; meanwhile, maintenance medium was provided as well. Each dilution was replicated 3 times, along with one control that contained no serum dilution. Plates were incubated for 5 days at 37°C and then stained with crystal violet, and the cytopathic effect (CPE) was evaluated. The titer of neutralizing antibodies was read as the highest dilution that gave complete protection.

### Virus-MAb binding assay.

The interaction of the viruses with purified neutralizing monoclonal antibodies (MAbs) was measured by ELISA. Briefly, 96-well plates were coated with 100 μl/well of inactivated EV71, CVA16, or VLPs (5 μg/ml in PBS buffer) at 4°C for overnight. The wells were then incubated sequentially with 100 μl/well of PBST (phosphate-buffered saline with 0.2% Triton X-100) plus 5% bovine serum albumin (BSA) at 37°C for 1 h and 100 μl/well of neutralizing MAb (D6, A9, or C33) at the dilutions indicated in Fig. 9 at 37°C for 1 h. Horseradish peroxidase (HRP)-conjugated goat anti-mouse IgG (Abnova) diluted (1:5,000) in PBST plus 1% BSA was used as the secondary antibody at 37°C for 1 h. Five washes with PBST were carried out between incubation steps. For color development, 100 μl/well of TMB (3,3′,5,5′-tetramethylbenzidine) mixture was added and incubated for 10 min, followed by the addition of 50 μl/well of 1 M H_3_PO_4_ to stop the reaction. Absorbance was measured at 450 nm in a 96-well plate reader.

### Analytical comparison of differences in surface structure.

To understand the extent of surface similarity between two structures (in this case CVA16 and EV71), we choose one structure as a reference structure. The second (test) structure is then optimally superposed on the reference structure, and for each solvent-accessible atom in the reference structure, the nearest solvent-accessible atom in the test structure is located. The distance between these two atoms is then stored in the B factor column of the reference structure. Coloring the reference structure surface by B factor then reveals the differences as regions with higher B factor values.

### Epitope prediction.

Structure-based *in silico* epitope predictions were carried out using the method described previously (36). It is a semiautomated procedure that uses crystallographic and noncrystallographic symmetries to generate a set of coordinates containing a central protomeric unit augmented by neighboring structures sufficient to ensure that epitopes spanning symmetry-related protomers can be identified as input for three freely available structure-based B cell epitope prediction programs, Epitopia (37), Discotope (38), and Ellipro (39). The consensus results from the three programs for the outer surface are then taken as potential epitopes.

## RESULTS

### Characterization and structure determination.

CVA16 (strain Ningbo.CHN/028-2/2009) was grown in Vero cells and formaldehyde inactivated prior to purification (see Materials and Methods). Mature virus and natural empty particles were separated by ultracentrifugation and characterized using SDS-PAGE, analytical ultracentrifugation, and the PaSTRy assay (40) as described previously (20). The sedimentation coefficients and λ260/λ280 absorbance ratios were ∼160S and 1.67 for the mature virus, and ∼80S and 0.64 for the empty particle, indicating that the latter does not contain RNA, and SDS-PAGE showed that VP0 is not cleaved into VP4 and VP2 in the empty particle. VLPs were secreted from Sf9 insect cells infected with recombinant baculovirus containing the P1 and 3CD genes of CVA16 (09-7 strain) and purified by ultracentrifugation. Diffraction data for inactivated CVA16 empty and full virus particles to 2.7- and 2.65-Å resolution, respectively, were collected at room temperature *in situ* (29). Crystals of CVA16 VLPs diffracted to 2.5 Å. The crystals of both mature and empty particles belong to the same space group, *P*4_1_2_1_2, with similar unit cell dimensions of *a* = *b* = 491 Å and *c* = 709 Å. There is one virus particle in the crystallographic asymmetric unit (60-fold NCS), positioned at (*1/4*, *1/4*, *1/4*). The space group of CVA16 VLP crystals is *P*4_2_32, with unit cell dimensions of: *a* = *b* = *c* = 347 Å. The particle is centered at the origin, and there is a pentamer in the crystallographic asymmetric unit (5-fold NCS). Structures were determined by molecular replacement with the mature EV71 (PDB accession number 3VBF) as a search model. Data collection and refinement statistics are shown in Table 1. Representative portions of the high quality electron density maps are shown in Fig. 1.

### The mature CVA16 particle is broadly similar to the mature EV71 particle.

**FIG 2:**
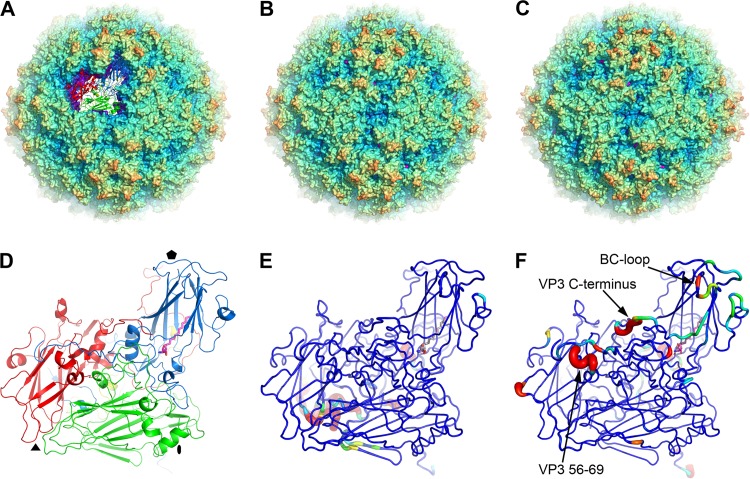
Overall structures. (A to C) Radius-colored surface representations of the mature CVA16, empty CVA16, and mature EV71 particles, respectively, cut away in panel A to highlight an individual protomer with proteins color coded as follows: VP1, blue; VP2, green; VP3, red; and VP4, yellow. (D) An enlarged protomer structure of the mature CVA16 as shown in panel A. The bound pocket factor in VP1 is drawn as magenta sticks. (E and F) Comparisons of the mature CVA16 protomer with those of empty CVA16 (E) and mature EV71 (F). Structural differences are mapped onto the protomer of the mature CVA16 virion; the thickness and color of the worm representation reflects the local deviation between the structures (from blue [≤0.5 Å] through green, to red [≥2.0 Å]). Regions missing in any particle are shown in red. The pocket factors are shown as gray sticks for empty CVA16 and mature EV71.

**FIG 3:**
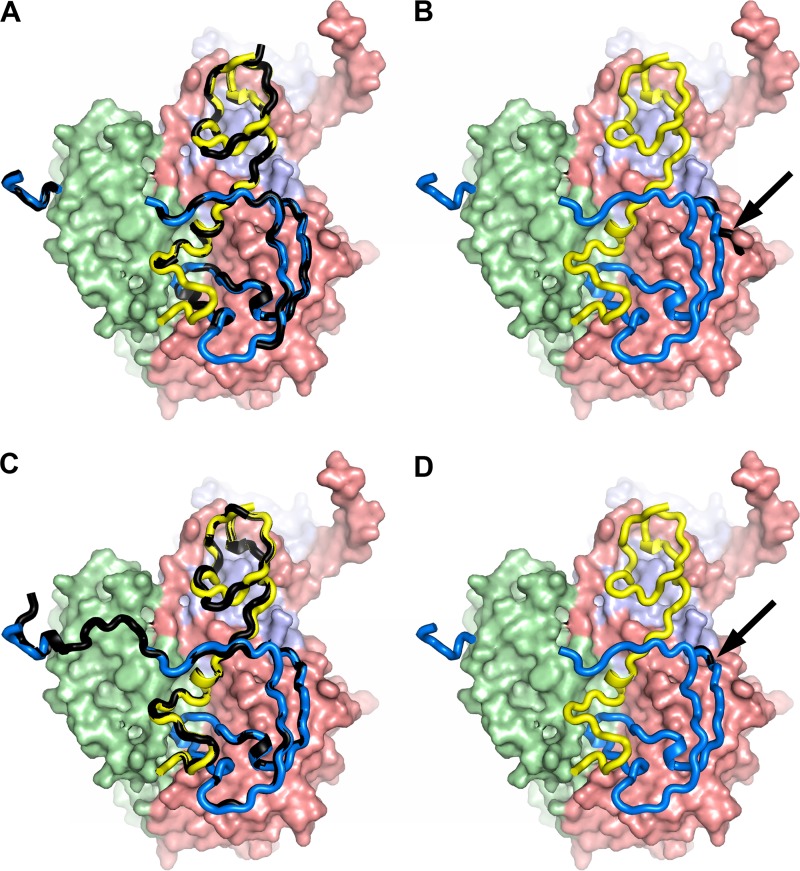
The structure of the particle inner surface. The N termini of VP1 and VP4 (blue and yellow ribbons, respectively) of mature CVA16 are compared with those (black) of empty CVA16 (A), expanded CVA16 (B), mature EV71 (C), and empty EV71 (D). VP1 (excluding the first 75 residues), VP2, and VP3 of mature CVA16 are shown as surface representations in pale blue, green, and red, respectively. Black arrows indicate where the N terminus of VP1 exits the capsid in the expanded 135S CVA16 (B) and the beginning of ordered structure in empty EV71 (D).

The mature CVA16 virus particle contains VP1 to -4 and is well ordered apart from residues 1 and 9 to 17 of VP1, 1 to 9 of VP2, and 1 to 11 of VP4. Given ∼80% identity (with no insertions or deletions) in the capsid protein sequences of CVA16 and EV71, it is unsurprising that their overall structures are very similar; 823 Cα out of 832 match with a root mean square deviation (RMSD) of 0.5 Å (Fig. 2) (3, 41). The most significant differences are on the outer surface, including the BC loop and C-terminal residues 280 to 297 of VP1 and, in VP3, residues 58 to 66 leading to the B strand and the C-terminal residues 233 to 239 (Fig. 2). Thus, residues 59 to 62 and 65 to 69 of VP3 form 2 short helices linked by a tight turn in CVA16, compared to a loop and a longer helix (62 to 69) in EV71 (Fig. 2F). The inner surface of the CVA16 particle is similar to that of EV71 and distinct from those of other known enteroviruses (Fig. 3): VP1 starts adjacent to the icosahedral 2-fold axis underneath the αA helix of VP2 and folds across the protomer, while residues 12 to 31 of VP4 form a loose spiral beneath VP1. VP1 contains a bound pocket factor, modeled as sphingosine (similar to EV71) (3).

### The natural empty particle is not expanded.

**FIG 4:**
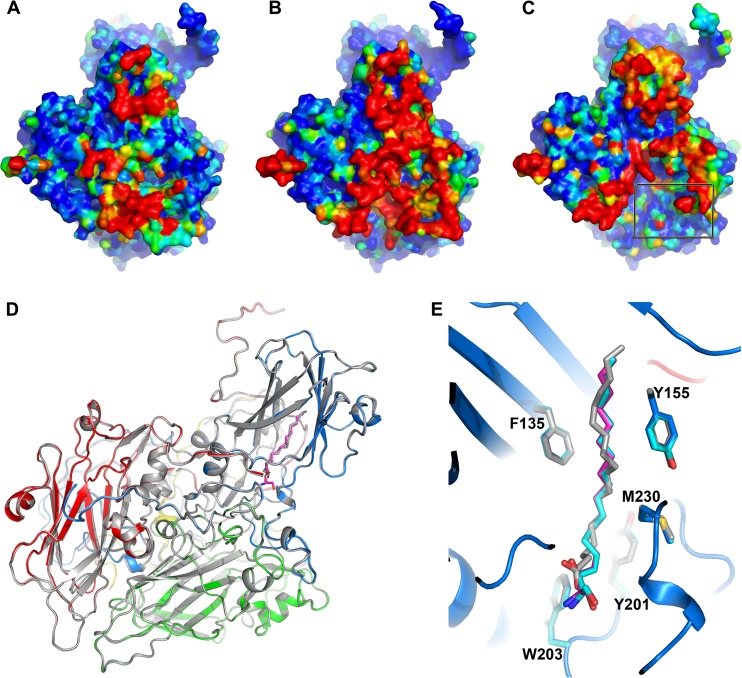
Comparison of the three CVA16 particle structures. (A to C) Protomer inner surfaces of the full, natural empty, and VLP particles, respectively, colored according to B factors (blue for the most rigid, through green, to red for the most flexible), showing that the internal structure of the VLP is more flexible than those of the full and empty natural particles and that more residues in the VP1 N terminus and VP4 are disordered (boxed area in panel C). (D) Ribbon diagram showing superimposed protomers of CVA16 full particle (colored as described in the legend to Fig. 2D) and VLP (gray). (E) Close-up view of the overlaid pocket factor binding sites in the full, natural empty, and VLP particles. For clarity, only the main chains of the full particle are shown as ribbons; the side chains are colored in blue, cyan, and gray and pocket factors in magenta, cyan, and gray for the full, empty, and VLP particles, respectively.

The natural empty particle contains proteins VP0, VP1, and VP3. Residues lining the inner surface of the particle are more flexible (with higher B factors) and some residues are essentially disordered (Fig. 4B), and thus, residues 9 to 18 and 39 to 42 in VP1 and 1 to 11 and 62 to 80 in VP0 (corresponding to 1 to 11 and 61 to 69 of VP4 and 1 to 9 of VP2 in the mature particle) are not defined in the electron density map (Fig. 3A). The overall structure is otherwise essentially indistinguishable from that of the mature virus (RMSD of 0.3 Å for 813 Cα atoms), and the pocket factor binding site is fully occupied by a moiety that again resembles sphingosine (Fig. 1B and 2E). However, the inner surface of the empty CVA16 particle is much more ordered than the expanded EV71 empty particle, where the entire 69 residues of VP4 and residues 1 to 72 of VP1 are invisible (Fig. 3D).

### The recombinant capsid is not expanded and harbors a different pocket factor.

The recombinant CVA16 capsid is self-assembled from VP0, VP1, and VP3. This particle is unexpanded and in the native antigenic state, and thus, the external surface of the VLP is structurally indistinguishable from the mature virus and natural empty particles (RMSD in Cα atoms of 0.4 Å and 0.3 Å, respectively) (Fig. 4). As expected, the residues lining the inner surface of the particle show more disorder; indeed, they seem a little more disordered than those of the natural empty particle (Fig. 4C). Disordered residues include residues 8 to 23 and 36 to 61 of VP1 and residues 1 to 13, 46 to 64, and 77 to 81 of VP0. Residues 65 to 76 of VP0, which include the cleavage site between 69 and 70 (cleavage of which produces VP4 and VP2) are well defined in the density. This region is structurally very similar to the corresponding region in immature poliovirus (42). There is a single residue change between the VLP and the natural particles, Thr240, located in the VP1 HI loop (Ile240 in the natural particle), but this does not introduce any significant structural change. The pocket factor binding site located inside the β-barrel of VP1 is fully occupied by a fatty acid acquired from the Sf9 insect cells, which differs somewhat from that derived from mammalian cells and is modeled as stearic acid (Fig. 1C and 4E). Compared to the head group in CVA16 mature and empty particles, the smaller carboxyl head group of the stearic acid is positioned about 3 Å lower than the hydroxyl oxygen of the sphingosine and forms a hydrogen bond to the main chain nitrogen of Leu113, similar to the carbonyl oxygen of the sphingosine (Fig. 4E).

### EV71 may have a greater propensity to expand than CVA16.

**FIG 5:**
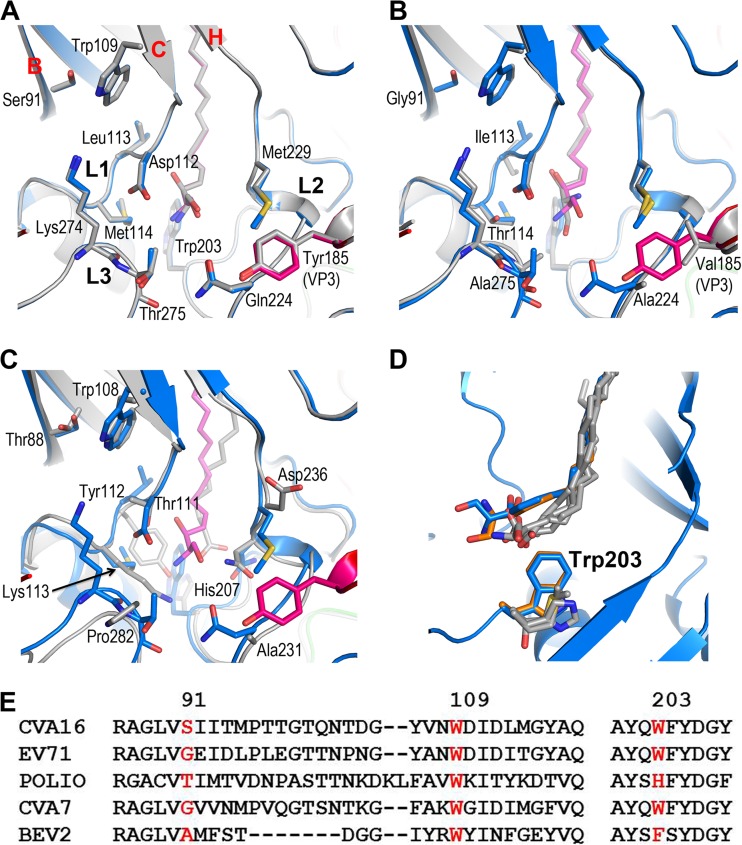
Structural differences around the VP1 pocket among enteroviruses. The structure of the mature CVA16 around the VP1 pocket is compared with those of empty CVA16 (A), EV71 (B), and poliovirus (C). The main chains are shown as ribbons, and the side chains as sticks with atom coloring. The mature CVA16 is colored as described in the legend to Fig. 2D, and the others in gray. Side chains of the mature CVA16 (A), EV71 (B), and poliovirus (C) are labeled. (D) Comparison of the positions of the bound pocket factors relative to residue Trp203 in CVA16 (blue), EV71 (orange), and poliovirus, bovine enterovirus 1, human rhinovirus 16, and swine vesicular disease virus (the latter four are in gray). (E) Sequence alignment of two VP1 regions of five enteroviruses. BEV2, bovine enterovirus 2.

The EV71 natural empty particles used in our earlier structural studies (produced by two independent companies) and those from a different strain used in another structural analysis were all in an expanded conformation (3, 43), while using identical methodology, those for CVA16 described here are not (although we have observed an expanded CVA16 intermediate [135S] particle [20]). Poliovirus type 1 (42) empty particles have also been observed to adopt an unexpanded conformation. Although the stability of unexpanded empty particles is rather finely balanced, it may be that those of CVA16 are slightly more stable than those of EV71. It is possible that the considerable residual order in the internal structural features of the unexpanded CVA16 empty particles is not present in EV71, and this might contribute to the difference. However, there is a suggestion from the structures that there may also be a contribution from the variable stability of pocket factor binding. Thus, the entrance of the VP1 pocket located on the floor of the canyon is composed of the βC-α4 loop (L1, residues 111 to 114), the adaptor-sensor region of the GH loop leading to the βH strand (L2, residues 224 to 230), and a tight turn leading to the long C-terminal loop (L3, residues 274 to 276) (Fig. 5A). Underneath the entrance to the pocket, which harbors the head group of the pocket factor, lies, in poliovirus, His207 (other viruses, e.g., bovine enterovirus 1, human rhinovirus 16, and swine vesicular disease virus, possess similar residues [44–46]) (Fig. 5C and D), whereas the equivalent residue in EV71 and CVA16 is Trp203. This positions the head group ∼4 Å closer to the virus surface than in poliovirus (42), increasing its solvent accessibility and, possibly, facilitating pocket factor escape and particle expansion (3, 20). However, in CVA16, the destabilizing effect may be mitigated by a series of small structural changes (Fig. 5B). First, Gly91 of βB is replaced by a serine in CVA16, making tighter van de Waals interactions with Trp109, together with more bulky residues at positions 113 and 114, stabilizing the βC and L1 loop (Fig. 5B). Second, residues 224 and 275 are replaced by bulkier residues in CVA16, making the pocket entrance smaller (Fig. 5B). Finally, Tyr185 (a valine in EV71) in the VP3 GH loop from an adjacent protomer packs against Gln224, stabilizing L3. These structural differences result in a 180° rotation of the sphingosine head group between EV71 and CVA16 (Fig. 5B). Since residues Gly91, Trp109, and Trp203 of EV71 are conserved in CVA7, we would expect these viruses to be similar (Fig. 5E).

### Receptor binding site.

**FIG 6:**
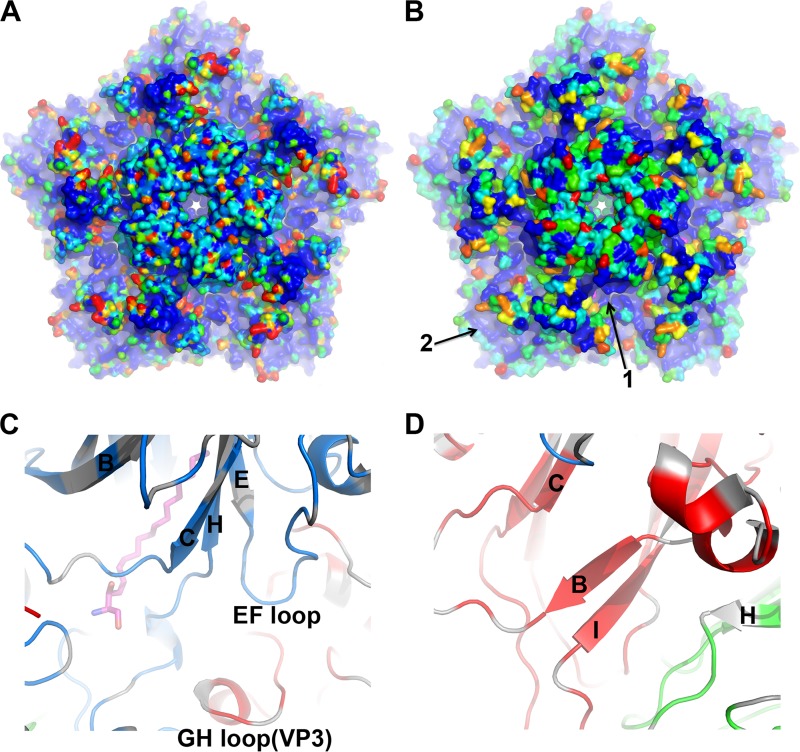
Conserved surface areas between CVA16 and EV71. (A) Surface shape variations between CVA16 and EV71 (see Materials and Methods) are colored from blue (≤0.5 Å) to red (≥3.0 Å) on a pentamer of CVA16. (B) Amino acid conservation between CVA16 and EV71 is mapped onto the surface of a CVA16 pentamer by rainbow coloring based on a conservation score (from 0.0 to 4.0). Ten strains of both CVA16 and EV71 from different regions were selected, and a conservation score for each residue was calculated using CONSURF (63). Two large conserved areas are indicated by arrows. (C and D) Ribbon diagrams show a closeup view of the structures of the two conserved areas labeled 1 (C) and 2 (D) in panel B. The color scheme is as described in the legend to Fig. 2D, and the strands of the β-sheet and loops are labeled according to convention.

Members of the HEVA subgenus can be divided into two major groups based on whether they require SCARB2 for infection, with EV71, CVA7, CVA14, and CVA16 using SCARB2. We have mapped both sequence conservation and similarity in surface shape (using a simple algorithm described in Materials and Methods) between EV71 and CVA16 onto the pentamer of the CVA16 capsid (Fig. 6), revealing two large conserved areas on the outer surface. The first is at the bottom of the canyon around the pocket entrance, comprising residues from strands βC and βH and the CD, GH, and EF loops of VP1 and the GH loop of VP3 from an adjacent protomer (Fig. 6C). The EF loop region of EV71 has been shown to be involved in interactions with SCARB2 (47), the VP1 and VP3 GH loops both undergo large conformation changes during uncoating (20), and SCARB2 can initiate uncoating (48, 49), suggesting that this area is involved in SCARB2 binding. The second conserved area, which lies mainly on VP3, close to the icosahedral 3-fold axis, includes a small conserved positively charged patch (Fig. 6D). The functional role of this site, if any, remains to be established.

### Antigenic variation between CVA16 and EV71.

**FIG 7:**
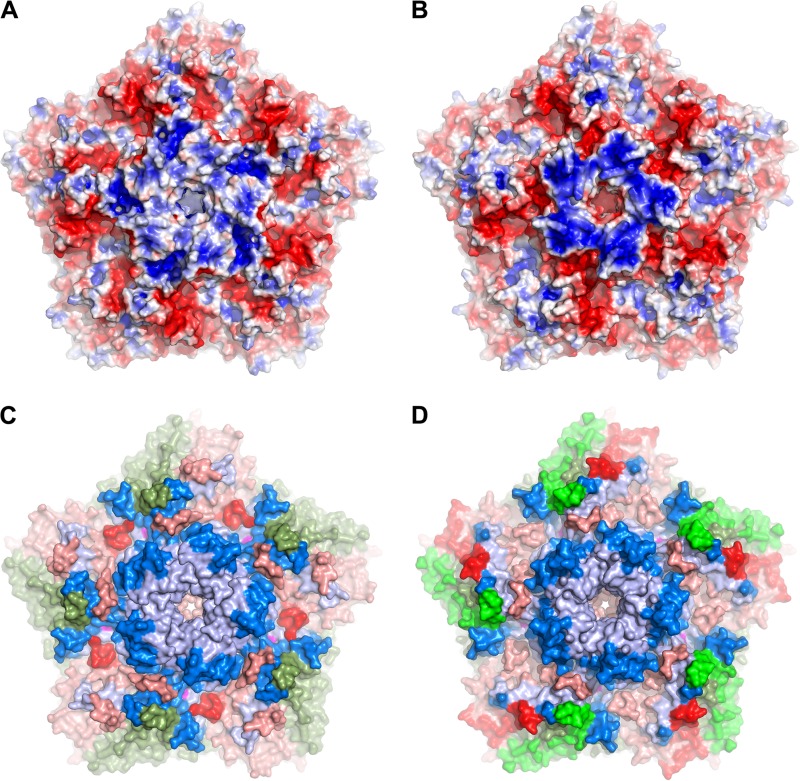
Antigenic sites. Electrostatic surfaces of CVA16 (A) and EV71 (B) displayed on pentamers of the respective structures. These were calculated using PYMOL; red represents a negative charge and blue a positive charge (±5 *kT*/*e*, where temperature [*T*] is equal to 298.15). (C and D) Epitopes of CVA16 (C) and EV71 (D). Surfaces of VP1, VP2, and VP3 are in pale blue, green, and red, respectively; epitopes are in bright colors.

**FIG 8:**
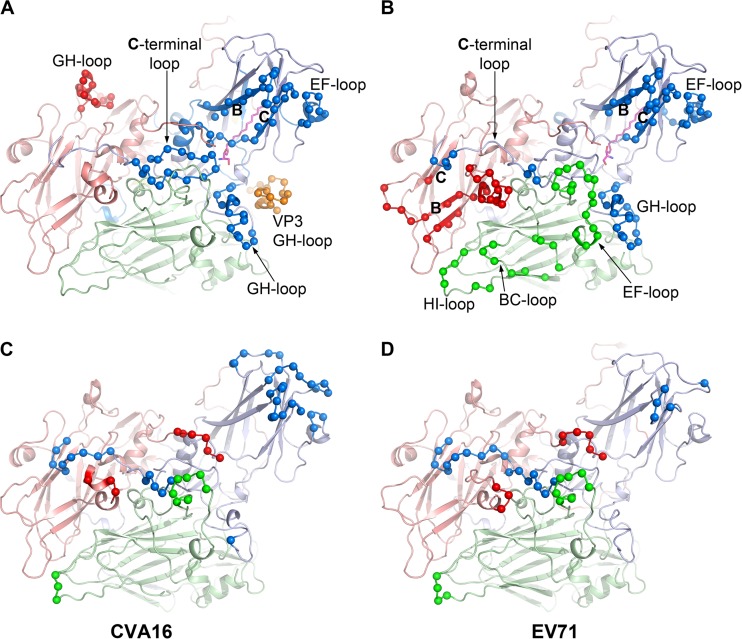
Experimental and predicted antigenic sites. (A and B) Antigenic sites for CVA16 (A) and EV71 (B). Capsid proteins are shown as ribbons in pale blue, green, and red for VP1, VP2, and VP3, respectively. Antigenic site residues are shown as small spheres in bright colors. The VP3 GH loop from an adjacent protomer in CVA16 is shown in orange in panel A. (C and D) Epitopes from *in silico* prediction for CVA16 (C) and EV71 (D) (see Materials and Methods).

EV71 vaccination does not induce cross protection against CVA16 (25–27), i.e., the neutralizing antibodies either do not bind or are incapable of neutralizing CVA16. This presumably arises from structural differences on the outer surface of the two viruses. Shape differences mapped onto CVA16 are shown in Fig. 6A, and the surfaces of the two viruses show similar electrostatic features: a positively charged patch around the 5-fold axis and a large negatively charged patch between the pocket entrance and the 2-fold axis (Fig. 7A and B). However, the positively charged area around the 5-fold axis in EV71 is smaller and more intense. We have mapped all known epitopes of the two viruses onto their pentamers (Fig. 7C and D) (50–59). These epitopes are concentrated on the walls of the canyon surrounding the proposed SCARB2 binding site, suggesting that neutralization might arise by steric occlusion of the receptor-binding site. However, a more powerful mechanism of neutralization would be to trigger conformational changes and initiate premature uncoating; indeed, the GH loop of VP1, the sensor adaptor region implicated in triggering uncoating, together with the EF loop residues 136 to 150 of VP2, forms one antigenic site in EV71 (3), and binding of MAb E18 to this epitope induces virus expansion and genome release (57). The VP3 GH loop in CVA16, which has not been reported to be an epitope in EV71, is close to the VP1 GH loop of an adjacent protomer and likely forms a single antigenic site with it (Fig. 8). Thus, the VP1 GH loop epitope employs a different structural component to form a distinct antigenic site in the two viruses. Clearly, the GH loop is not the only neutralizing epitope in EV71, since a chimeric EV71 VLP in which the VP1 GH loop is replaced by that of CVA16 can generate protection against EV71 in mice (60). It would be interesting to know if a chimeric EV71 VLP containing both VP1 and VP3 GH loops of CVA16 induces stronger immunogenicity against CVA16.

**FIG 9:**
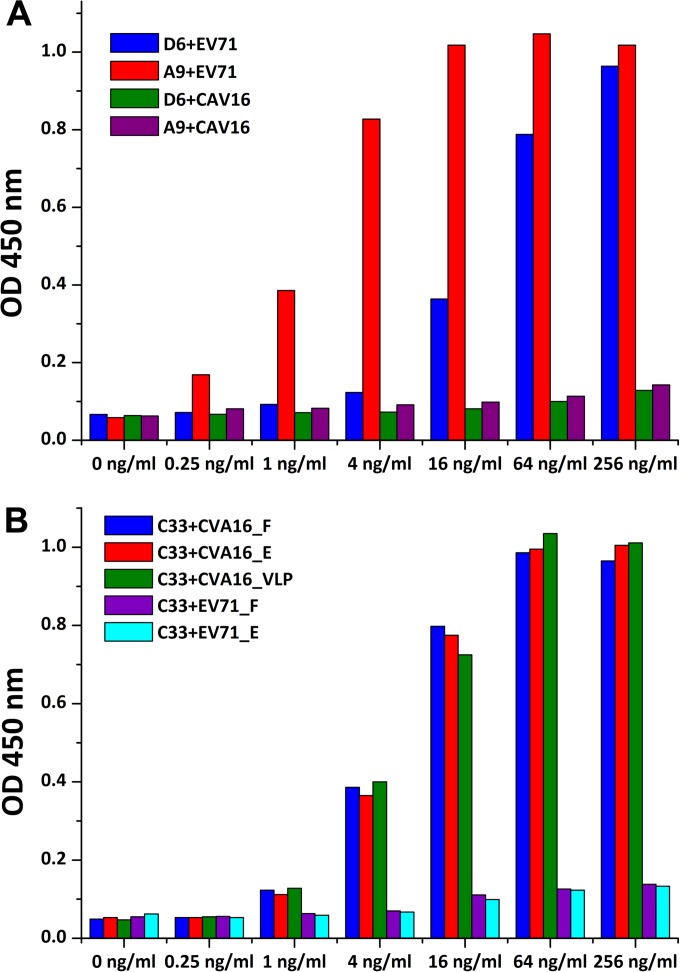
Virus-MAb binding assay. The reactivity of EV71 and CVA16 particles against a panel of three purified neutralizing monoclonal antibodies at the indicated dilutions was measured by ELISA. (A) Ability of neutralizing monoclonal antibodies D6 and A9 to bind EV71 and CVA16 particles. (B) Ability of neutralizing monoclonal antibody C33 to bind CVA16 full and empty particles and VLPs and EV71 full and empty particles.

To explore further why EV71 vaccines do not protect against CVA16 and whether CVA16 virus neutralizing antibodies can cross-neutralize EV71, two MAbs capable of neutralizing EV71, D6 and A9, and one neutralizing MAb against CVA16, C33, were generated (see Materials and Methods and Table 2). D6 and A9 have no cross-neutralizing activity against CVA16, and C33 could not protect against EV71 infection. Furthermore, D6 and A9 do not bind to CVA16 and C33 does not bind EV71 (Fig. 9). CVA16 full and empty particles and VLPs all exhibit similar levels of binding affinity to C33 (Fig. 9), as expected, since their surface structures are indistinguishable. In line with this, it has been demonstrated that CVA16 VLPs are able to induce neutralizing activity against CVA16 infection in mice (28).

**TABLE 2:** *In vitro* neutralization assays of monoclonal antibodies against EV71 and CVA16

Antibody	Neutralization titer for:	
	EV71	CVA16
D6	1:512	<1:8
A9	1:2048	<1:8
C33	<1:8	1:128
HAV MAb no. 7^*a*^	<1:8	<1:8

a Human hepatitis A virus MAb number 7 was used as a negative control.

Finally, we have used an *in silico* epitope prediction method (36) on both viruses (Fig. 8). As expected, the predicted epitopes map broadly onto those observed, with the exception that the EF loop of VP2 is observed as an epitope only in EV71, although it is predicted as one in both viruses. It is likely that this reflects incomplete mapping of the antigenic surface of CVA16, which is less well characterized. However, one notable feature is that the major neutralizing region around the receptor binding site (including the VP3 GH loop) is underrepresented in the *in silico* predictions, and we suggest that this might reflect both the slightly recessed nature of this region of the capsid and the fact that methods like peptide mapping, which pick out neutralizing epitopes, may naturally focus on highly neutralizing regions, such as those regions where receptors bind and induce expansion of the capsid.

## DISCUSSION

We have determined structures for CVA16 mature particles and natural empty capsids, as well as recombinant empty particles produced in insect cells. The outer surfaces are indistinguishable and interact similarly with monoclonal antibody, in line with recombinant CVA16 particles eliciting strong protection in mice (61). Capsid expansion to an antigenically distinct form is a prerequisite for the release of RNA and is usually triggered by receptor engagement. However, an equilibrium exists between at least partially expanded particles and mature capsids, and the balance of this will vary between viruses. We suggest that a particular residue (Trp203) in the VP1 pocket of CVA16 and EV71 (Fig. 5) may explain why the EV71 natural empty particle expands more easily, whereas CVA16 partially mitigates this effect. Such information may facilitate the production of more robust unexpanded particles, which could be used as a vaccine antigen. It is also possible that the considerable residual order in the internal structural features of the unexpanded CVA16 empty particles stabilizes them. The substantial overlap of antigenic sites between the two viruses, which are nonetheless not cross-reactive, suggests that a bivalent vaccine might be required to produce a broadly effective, safe HFMD vaccine. In addition, the structures reported may facilitate structure-based design of potent inhibitors (62) to combat HFMD directly or to replace the natural pocket factor to produce a more stable VLP vaccine.

We have identified two conserved patches on the virus surface and proposed the area around the VP1 pocket entrance to be the SCARB2 receptor binding site, in agreement with reports that the VP1 EF loop is crucial for SCARB2 binding (47, 48). This area includes the VP1 GH loop, likely to be critical for cellular receptor attachment, and the VP3 GH loop, which undergoes the largest conformational changes during virus uncoating (3, 20), in line with the finding that SCARB2 is capable of inducing viral uncoating, rather than PSGL-1 (24). Since there is a fundamental commonality of receptor binding for the two viruses, we suggest that cross-reactive antibodies might possibly be enriched if the immune response could be more narrowly focused on these regions.
